# CTL-Derived Granzyme B Participates in Hippocampal Neuronal Apoptosis Induced by Cardiac Arrest and Resuscitation in Rats

**DOI:** 10.3389/fneur.2019.01306

**Published:** 2019-12-12

**Authors:** Ning-Ning Ji, Liang Wu, Bo-Ming Shao, Qing-Xiang Meng, Jin-Nan Xu, Hao-Wen Zhu, Yong-Mei Zhang

**Affiliations:** ^1^Jiangsu Province Key Laboratory of Anesthesiology, Xuzhou Medical University, Xuzhou, China; ^2^Anesthesiology Department of the First People's Hospital of Xuzhou, Xuzhou, China; ^3^School of Anesthesiology, Xuzhou Medical University, Xuzhou, China

**Keywords:** cardiac arrest, cardiopulmonary resuscitation, hippocampus, cytotoxic T lymphocytes, granzyme B, apoptosis

## Abstract

Hippocampal neuronal apoptosis is a devastating consequence of cardiac arrest (CA) and subsequent cardiopulmonary resuscitation (CPR). In this study, we assessed the contribution of cytotoxic T lymphocyte (CTL)-derived toxic mediator granzyme B (Gra-b) to the hippocampal neuronal apoptosis following CA/CPR in rats. Rats that experienced CA/CPA presented with cytosomal shrinkage, dense cytoplasm, and intensive eosinophilic staining in the CA1 region of dorsal hippocampus. CA/CPR rats also exhibited inability in spatial navigation and a local infiltration of peripheral CD8+ T cells into the hippocampus. The protein levels of Gra-b, cleaved Caspase-3, and cleaved PARP1 were significantly elevated in rats undergoing CA/CPR. Pretreatment with Gra-b inhibitor suppressed Gra-b release, attenuated hippocampal neuronal apoptosis, as well as improved cognitive impairment. Together, this study indicates that CTL-derived Gra-b is involved in the CA/CPR-induced neuronal apoptosis, and pharmacological manipulation of Gra-b may represent a novel avenue for the treatment of brain injury following CA/CPR.

## Introduction

Cardiac arrest (CA), an abrupt loss of heart function in individuals with or without heart disease, is a leading cause of cardiovascular and cerebral hypoxia and ischemia, resulting in disability and mortality (1). Despite the application of cardiopulmonary resuscitation (CPR) to reduce the mortality from CA, brain ischemia remains a devastating complication of CA/CPR, with considerable neuronal apoptosis and cognitive deficits within the initial 3 months after CPR in ~70% of the survivors (2, 3). To date, there is a paucity of approaches with clinical efficacy, reliability, and validity to prevent brain injury following CA/CPR (4).

Sustained CA and hypoxia induced ischemic brain injury with the onset of secondary neuroinflammation, including glial activation, peripheral immune cell recruitment, and pro-/anti-inflammatory factor release, resulting in pyrexia, hypophagia, hyperalgesia, and cognitive dysfunction (5). Meanwhile, the inflammatory responses facilitate cell fragment clearance, tissue repair, and functional recovery (6). In addition, studies of the central nervous system (CNS) evidenced an elevation of CD8+ cytotoxic T lymphocytes (CTLs) in a number of pathogen-induced neurological disorders (7–9), wherein activated CTLs can generate a series of serine proteases, particularly granzymes (Gra), which can induce cellular apoptosis, leading to neuronal death in ischemic brain regions (10–12). Moreover, Gra-b reportedly participates in the development of neurological diseases, such as brain ischemia-reperfusion injury (13). Despite the *de facto* infiltration of CTLs into the CNS after CA/CPR, the precise contributions of CTL-derived Gra-b to neuronal apotosis remain elusive. Herein, we aimed to investigate the effects of CTLs-derived Gra-b on the modulation of hippocampal neuronal apoptosis in a rat model of CA.

## Materials and Methods

### Animals

Adult male Wistar rats (weighing 250–300 g) were purchased from Jining Lukang Animal Co. Ltd. (Shandong, China) and housed in a 12 h light and dark cycle (lights on at 7:00 a.m.) with *ad libitum* access to food and water. All experimental protocols were approved by the Institutional Animal Care and Use Committee of Xuzhou Medical University [SYXK (Su) 2010-2011].

### Experimental Procedures

Rats were mainly randomized into sham (*n* = 15), CA/CPR (*n* = 20), and Gra-b inhibitor (*n* = 20) groups according to random number table. The CA/CPR model was established by asphyxia-induced CA and subsequent CPR as described previously (14). In brief, the ventilator connected with trachea was disconnected to induce hypoxic CA in anesthetized rats. CPR was implemented by manual precordial compressions and mechanical ventilation after 6 min of untreated cardiac arrest. Manual precordial compressions were maintained at a rate of about 200 per minute. Compression depth was ~30% of anteroposterior chest diameter at maximal compression. Ventilation was resumed by a volume controlled small animal ventilator with a frequency of 100 breaths-per-minute, an inspired O_2_ fraction of 1.0 and a tidal volume of 6 ml/kg. Ventricular fibrillation, if appropriate, was removed with up to three 2-J shocks after 8 min of CPR. If restoration of spontaneous circulation (ROSC) was not achieved, a 30 s interval of CPR was performed before a subsequent sequence of up to 3 shocks was attempted. This procedure was repeated for a maximum of three cycles. ROSC was defined as a return of supraventricular rhythm with a mean aortic pressure above 50 mmHg for a minimum of 5 min. In the case of spontaneous respiration, the ventilator was powered off. Gra-b inhibitor I (0.5 mM/kg body weight; 368050, Calbiochem, USA) was administered immediately after ROSC via a femoral catheter. Sham group only received identical surgical procedures except asphyxia.

### Cerebral Performance Category (CPC) and Morris Water-Maze (MWM)

The neurological deficiency score after CA/CPR was recorded for 4 consecutive days according to CPC scoring system in which scores range from 0 to 5 based on consciousness, motor function, and sensory function. 0 stands for normal status, 1 for mild cerebral disability, 2 for moderate cerebral disability, 3 for severe cerebral disability, 4 for coma/vegetative state, and 5 indicates brain death. Morris water-maze testing was conducted as described previously (15).

### Blood-Brain Barrier Permeability

Blood-brain barrier (BBB) permeability was detected by measurement of the Evans blue (EB) extravasation (16). EB dye (4% in 0.9% saline) was injected into the caudal vein (4 mL/kg). Two hours afterwards, rats were transcardially perfused prior to the isolation of ischemic hemisphere. The EB level in brain tissue was determined by spectrophotometry at a wavelength of 660 nm.

### Hematoxylin and Eosin Staining

Three days after CA/CPR, rats were transcardially perfused with 0.9% saline followed by 4% paraformaldehyde under deep anesthesia. Rat brains were isolated and post-fixed with paraformaldehyde for another 24 h prior to embedment in paraffin. The rat hippocampus was coronally sliced at 4 μm and stained with hematoxylin and eosin.

### Western Blotting

Rat hippocampus was homogenized in RIPA lysis buffer, with 50 μg protein sampled for SDS-PAGE. After the protein transference, the PVDF membrane was rinsed in washing buffer for 5 min, followed by addition of 5% skim milk powder, at room temperature (r/t) for 2 h. Blots were then incubated in anti-β-actin (1:1,000, rabbit, Sigma-Aldrich), anti-Gra-b (1:200, rabbit, Abcam), anti-caspase 3 (1:200, rabbit, Santa Cruz), or anti-PARP1 (1:200, rabbit, Santa Cruz) overnight at 4°C. On the following day, the PVDF membranes were maintained at r/t for 30 min, and were thereafter rinsed with the washing buffer for 10 min in triplicate, followed by incubation with anti-rabbit IgG with alkaline phosphatase (1:1,000, A0208, Beyotime, China) on the shaking table at r/t for 2 h. The blots were developed by nitro-blue tetrozolium/bromochbating in alkaline phosphate (NBT/BCIP) substrate after incubation in alkaline phosphatase-conjugated secondary antibodies for 2 h at r/t. ImageJ software was employed for grayscale analysis.

### Immunofluorescence Analyses

The hippocampus was sliced at 30 μm thickness with a cryostat (Leica CM1800; Heidelberg, Germany). For immunofluorescence staining, slices were blocked by 10% goat serum for 120 min and incubated with primary antibodies, including anti-CD-8 (1:100, mouse, Abcam), anti-TCR (1:100, mouse, Abcam), anti-Gra-b (1:100, rabbit, Abcam), anti-NeuN antibody (1:50, rabbit, CST; 1:50, mouse, Millipore), anti-Annexin V antibody (1:100, goat, Proteintech) for 24 h at 4°C following by incubation in secondary antibodies for 1 h at 37°C and mounted with 90% glycerol. The sections were visualized by a Leica confocal microscope. ImageJ Pro software was adopted for cell quantification.

### Statistical Analysis

All data are expressed as mean ± SEM. CPC scores and MWM acquisition data were analyzed by two-way repeated measures analysis of variance (ANOVA) followed by Tukey's *post-hoc* multiple comparisons. The number of CD8+ cells and apoptosis cells were analyzed by unpaired Student's *t*-test. The data of WB assays were analyzed by one-way ANOVA followed by *post-hoc* Student's Newman–Keuls (SNK) test. *p* < 0.05 was considered statistically significant.

## Results

There was no significant difference in the survival rate at 24 h after resuscitation between the CA/CPR and inhibitor groups (12/20 vs. 12/20; *P* > 0.05). Then half of the rats resuscitated successfully from cardiac arrest were used for immunofluorescence staining and HE staining (*n* = 6), and the rest of the rats were used for western blotting (*n* = 6).

### CA/CPR-Induced Hippocampal Neuronal Apoptosis

**Figure 1:**
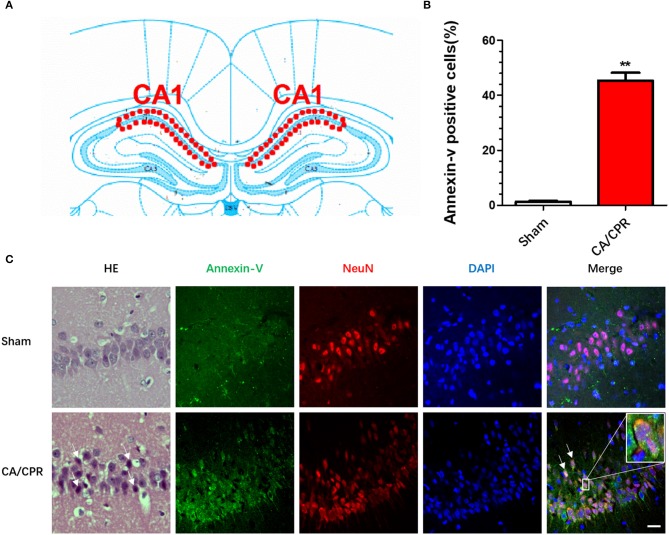
CA/CPR induced hippocampal neuronal apoptosis. The immunofluorescent staining was conducted in the CA1 region of dorsal hippocampus 24 h after CA/CPR. **(A)** Diagram of hippocampus. **(B)** The percentage of Annexin V^+^ cells in total NeuN^−^ cells was calculated. CA/CPR rats presented with a significant increase in the Annexin V^+^ cells as compared to the sham rats. **(C)** The H-E staining showed that cytosomal shrinkage, dense cytoplasm, and intensive eosinophilic staining of the neurons, and the immunofluorescence assay revealed the expression of hippocampal Annexin V, a protein marker of early-stage apoptosis, was significantly increased as compared to that in the sham group. The hippocampal neurons were labeled with NeuN, a neuronal marker. Scale bar = 20 μm. Data are expressed as mean ± SEM; *n* = 6; ^**^p < 0.01.

The interval between asphyxia and arrest was usually 5–6 min. Spontaneous circulation (ROSC) was restored about 1 min after CPR. The rats were subjected to CA/CPR, and were euthanized 24 h thereafter, with the CA1 region of dorsal hippocampus isolated and assessed by H-E staining and immunofluorescence labeling of Annexin V (Figure 1A), respectively. The H-E staining indicated cytosomal shrinkage, dense cytoplasm, and intensive eosinophilic staining of the neurons, indicating neuronal apoptosis. In parallel, the immunofluorescence assay revealed the expression of hippocampal Annexin V, a protein marker of early-stage apoptosis, was significantly increased as compared to that in the sham group (Figure 1C). The Annexin V-positive cells in the CA1 pyramidal cell layer in the dorsal hippocampus from CA/CPR rats significantly multiplied as compared to the sham rats (24.67 ± 0.88 vs. 7 ± 1.53; *t*_(4)_ = −10.02, *p* = 0.001; Figure 1B).

### Treatment With Gra-b Inhibitor Alleviated CA/CPR-Induced Behavioral Impairment

**Figure 2:**
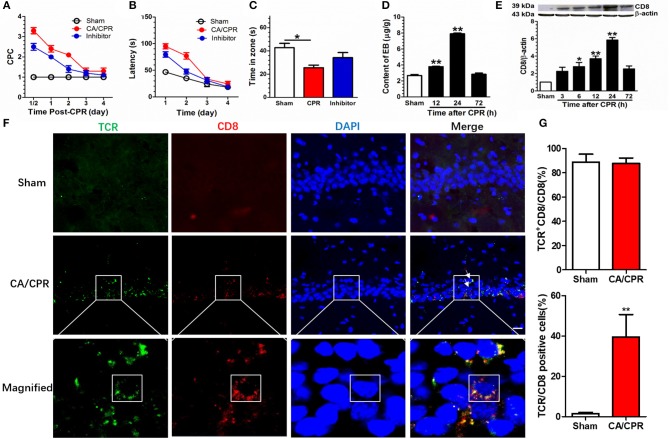
Gra-b inhibitor alleviated CA/CPR-induced behavioral impairments. **(A)** CA/CPA increased the CPC score (CPR vs. Sham, *p* < 0.01) and Gra-b inhibitor suppressed the increase in CPC score (CPR vs. inhibitor, *p* < 0.01). **(B)** CA/CPA impaired the spatial learning (CPR vs. Sham, *p* < 0.01) and Gra-b inhibitor alleviated the impairment in spatial navigation (CPR vs. inhibitor, *p* = 0.01). **(C)** CPR in rats reduced the duration in target zone (*p* = 0.012), which was attenuated by Gra-b inhibitor. **(D)** The CA/CPR rats exhibited an increase in BBB permeability as demonstrated by the brain EB level at 12 and 24 h after CA/CPR. **(E)** Western blot assay showed increased CD8 protein level at 6, 12, and 24 h after CPR (all *p* < 0.05) **(F,G)** CA/CPR rats displayed an increase in hippocampal CD8 staining, dominantly co-localized with the T Cell marker T cell receptor (TCR) (88%), and CD8/TCR-positive cells accounted for 36% of the cell population (CPR vs. Sham, *p* < 0.01). The white square in the lowermost immunofluorescence diagram showed that the CD8/TCR-positive cells were approximately circular, with significant difference from the typical hippocampal pyramidal neurons. Scale bar = 20 μm. Data are expressed as mean ± SEM; *n* = 6; ^*^p < 0.05, ^**^p < 0.01.

The CA/CPR rats displayed shuffling gait, decline in consumption of water and food, as well as neurologic and cognitive deficits. Administration of Gra-b inhibitor abated the cerebral performance category (CPC) scores in CA/CPR rats. A two-way repeated measures ANOVA revealed significant main effects in duration (*p* < 0.001), manipulation mode (*p* < 0.001), and significant duration × manipulation interaction (*p* < 0.001). *Post-hoc* Bonferroni multiple comparisons showed that elevated CPC scores in CA/CPR rats (CPR vs. Sham, *p* < 0.001) and Gra-b inhibitor annulled the elevation of CPC scores (CPR vs. inhibitor, *p* < 0.001; Figure 2A).

Gra-b inhibitor improved the spatial navigation performance in a Morris water maze (MWM) task in CA/CPR rats. A two-way repeated measures ANOVA revealed significant main effects in duration (*p* = 0.000), manipulation (*p* < 0.001), and significant duration × manipulation interaction (*p* < 0.001). *Post-hoc* Bonferroni multiple comparisons showed that CA/CPA impaired the spatial learning ability (CA/CPR vs. Sham, *p* < 0.001) and Gra-b inhibitor alleviated the impairment in spatial navigation (CA/CPR *vs*. inhibitor, *p* = 0.01; Figure 2B).

Gra-b inhibitor improved the retrieval of spatial memory in an MWM task in CA/CPR rats. A one-way ANOVA indicated a significant difference (*p* = 0.014; Figure 2C). *Post-hoc* Bonferroni multiple comparisons showed reduced duration in target zone for CA/CPR rats (*p* = 0.012), which was reversed by Gra-b inhibitor.

The blood-brain barrier (BBB), in integrity, plays a protective role to the organism, whereas its disintegration leads to the infiltration of peripheral immune cells via the site of lesion. Hence, the permeability of BBB following CA/CPR was evaluated by Evans Blue (EB) assay 24 h after CA/CPR. The EB concentration was significantly higher in the CA/CPR group than in the sham group, which demonstrated that CA/CPR increased BBB permeability (*p* < 0.001, Figure 2D). *Post-hoc* Bonferroni multiple comparisons showed an increase in EB level at 12 and 24 h after CA/CPR (all *p* < 0.01). Thereafter, a multitude of CD8 T cells were identified to have infiltrated into hippocampus. In parallel, Western blotting assay showed upregulated expression of the CD8 protein in CPR group (*p* < 0.001). *Post-hoc* Bonferroni multiple comparisons showed the increased CD8 protein at 6, 12, and 24 h after CPR (all *p* < 0.05; Figure 2E). Additionally, the CD8 signal was predominantly co-localized with the T cell marker T cell receptor (TCR) (88%) and CD8/TCR-positive cells accounted for 36%, based on immunofluorescence staining (Figures 2F,G).

### CA/CPR-Induced Elevation of Hippocampal Gra-b Level

**Figure 3:**
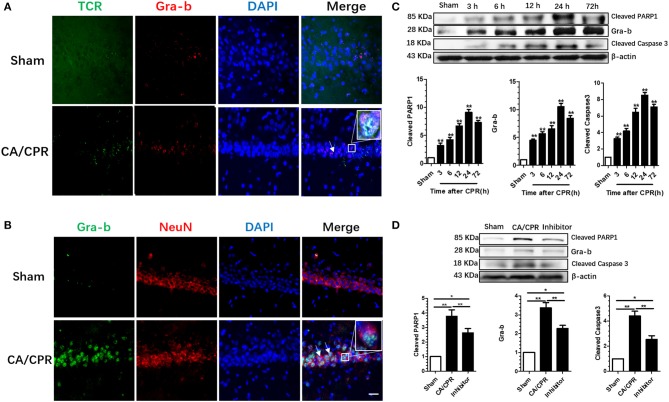
Gra-b inhibitor suppressed the increase in CA/CPR-induced apoptotic factors. **(A)** CA/CPR rats presented with an increase in Gra-b protein expression and co-localization with TCR. **(B)** Numerous Gra-b interacted directly with neurons in CPR group. **(C,D)** Gra-b level was increased at 12 and 24 h after CPR. Cleaved Caspases-3 level was increased at 3–72 h after CPR. Cleaved PARP1 level was increased at 12–24 h after CPR. CPR induced an increase in Gra-b level, which could be annulled by Gra-b inhibitor. CPR induced an increase in cleaved Caspase-3 level, which could be reversed by Gra-b inhibitor. CPR induced an increase in cleaved PARP level, which could be annulled by Gra-b inhibitor. Scale bar = 20 μm. Data are expressed as mean ± SEM; *n* = 6; ^*^p < 0.05, ^**^p < 0.01.

The expression of hippocampal Gra-b was assessed to explore the role of CD8+ CTL in CA/CPR-induced brain injury. Immunofluorescence staining revealed that TCR signal was dominantly co-localized with the Gra-b protein, suggesting that CD8+ CTL serving as the origin of Gra-b protein (Figure 3A). CA/CPR rats exhibited an increase in Gra-b protein expression and co-localization with NeuN (Figure 3B). CA/CPR upregulated the Gra-b protein expression as demonstrated by a one-way ANOVA (*p* = 0.003). *Post-hoc* Bonferroni multiple comparisons revealed the increased Gra-b level after CPR (all *p* < 0.05; Figure 3B).

CA/CPR upregulated the cleaved Caspase-3 protein expression as validated by a one-way ANOVA (*p* < 0.001; Figure 3C). *Post-hoc* Bonferroni multiple comparisons showed the increased level of cleaved Caspase-3 as from 3 to 72 h after CPR (all *p* < 0.01; Figure 3C). CA/CPR upregulated the cleaved PARP1 protein expression as demonstrated by a one-way ANOVA (*p* = 0.042; Figure 3C). *Post-hoc* Bonferroni multiple comparisons showed the increased level of cleaved PARP1 protein after CPR (all *p* < 0.05; Figure 3C).

Gra-b inhibitor is well-documented to modulate apoptosis, which would incite interest to ascertain whether and how the apoptosis-signaling pathway in the rat brain was involved in this process. Western blotting was employed to detect the variations of protein levels of Gra-b, cleaved Caspase-3, and cleaved PARP1. One-way ANOVA revealed that Gra-b inhibitor significantly reversed the upregulation of Gra-b (*p* < 0.001), cleaved Caspase-3 (*p* < 0.001), and cleaved PARP1 (*p* < 0.001), respectively (Figure 3D).

## Discussion

Our present study confirmed that CA/CPR could increase the permeability of BBB, allowing blood CD8+ CTLs to infiltrate into the brain tissue and secrete Gra-b protein, and leading to neuronal apoptosis and death, probably via the Caspase-3 signaling pathway. Administration of Gra-b inhibitor annulled the upregulation of Gra-b, cleaved Caspase-3, and cleaved PARP1 levels, thus ameliorating neuronal apoptosis as well as behavioral and cognitive impairment.

The brain was previously thought to be an immune-privileged organ, containing resident immune cells that maintain the homeostasis of central nervous system. Recent studies demonstrated that the CNS has a canonical functional lymphatic system or meningeal lymphatic vessels, which directly connect deep cervical lymph nodes and promote the entry of immune cells into the brain (17, 18). Notwithstanding BBB prevents the immune cells from entry into the CNS, ischemic injury-induced BBB leakage allows peripheral immune cells to infiltrate into the brain parenchyma, and activate the resident microglia and astrocytes, or induce neuronal death via the direct action on the nervous system, as illustrated by the ability of CD8+ T cells to detect and destroy the MHC I-positive neurons (16).

The entry of blood T cells into the CNS may induce a delayed injury (19, 20). In the present study, CA/CPR could induce the infiltration and activation of CD8+ CTLs, with a scant population of glial cells existent in the hippocampus after CA/CPR (Supplementary Material). The peak levels of CD8+ CTLs in the hippocampus occurred at 24 h after CA/CPR. CA/CPR can induce brain ischemia and related adverse responses, e.g., Ca^2+^ overload, mitochondrial dysfunction, ATP depletion, pro-apoptotic protein expression, and glutamate excitotoxicity (21–23). Furthermore, CA/CPR can provoke inflammation, initiate immune responses, and induce cell death, which contributes to neurologic deficit and vulnerability of CA1 pyramidal neurons of the hippocampus to ischemic brain injury (16, 24).

The CD8+ CTLs can reportedly destroy target cells by the secretion of Gra-b or by contact. CD8+ knockout CTLs have been shown to decrease the infarcted area. Gra-b, a member of serine protease family, is a potent inducer of caspase-dependent and independent forms of apoptosis. Once released by T cells and NK cells, Gra-b can directly cleave several substrates or pro-apoptotic proteins, e.g., PARP, to induce cell death (25). In addition, Gra-b is also capable of cleaving Bid into its truncated form (tBid), which subsequently migrates to the outer mitochondrial membrane to interact with Bcl-2, and induce the release of Bad and Bax, with initiation of the intrinsic cell death pathway. Gra-b preferentially cleaves pro-caspases to initiate cell death in rats (26–30). PARP is a DNA-repair enzyme, cleaved at aspartame residues to 89 and 21 kDa signature fragments by caspase during apoptotic cell death. Gra-b is involved in the neuronal degeneration in stroke in humans and neuronal death in cerebral ischemia in rats (13). In addition, inhibition of Gra-b could attenuate CD8 T cell-induced neurotoxicity and neuronal death. The present study revealed that the levels of Gra-b, cleaved caspase-3, and cleaved PARP1 increased at 3 h and reached the peak levels at 24 h. Inhibition of Gra-b expression resulted in the downregulation of the expression of Gra-b, cleaved caspase-3, and cleaved PARP1, with the apoptosis of hippocampal CA1 neurons mitigated and neurological function improved. Hence, our results indicated that infiltration of T cells into the brain induced the secretion of Gra-b, contributing to neuronal apoptosis and ultimately,neuronal destruction.

Our study has limitations. The duration of brain injury after heart failure is a long process, whereas this study only focused on changes within the initial 72 h after CA/CPR. Animal experiments can only simulate instead of being equivalent to clinical scenario, and further studies which could extend the observation duration to verify the role of CTL-derived Gra-b in the process and prognosis of brain injury.

In summary, our current study evaluated whether CTL-derived Gra-b participated in neuronal apoptosis and death after CA/CPR. It is plausible that Gra-b induced neuronal apoptosis and death by activating caspase-3 and cleaving PARP1. The Gra-b inhibitor could suppress neuronal apoptosis and death, protect neuronal integrity, and improve the outcome. These data indicate Gra-b may be a key contributor of neuronal apoptosis and functional impairment after CA/CPR. Thus, we might postulate that modulation of Grab expression could attenuate the neuronal damages and benefit prognosis of CA/CPR. For the paucity and inefficacy of regimens for secondary brain injury after CA/CPR, suppression of Gra-b activity by pharmacological strategy may pave a novel avenue to therapeutics for CA/CPR -induced brain injury.

## Data Availability Statement

All datasets generated for this study are included in the article/supplementary material.

## Ethics Statement

The animal study was reviewed and approved by the Institutional Animal Care and Use Committee at Xuzhou Medical University.

## Author Contributions

N-NJ contributed to the experimentation, data collection, and manuscript composition. LW, B-MS, J-NX, and H-WZ helped experiment performance. Q-XM assisted in data collection. Y-MZ contributed to the conception and supervision of the research, data interpretation, manuscript drafting, and revision. All authors have read and approved the submission and publication of the final version of manuscript. The authors vouch for the accuracy and completeness of the experiment.

### Conflict of Interest

The authors declare that the research was conducted in the absence of any commercial or financial relationships that could be construed as a potential conflict of interest.
